# Flexible bronchoscopy may decrease respiratory muscle strength: premedicational midazolam in focus

**DOI:** 10.1186/2049-6958-7-31

**Published:** 2012-09-25

**Authors:** Baykal Tulek, Fikret Kanat, Sule Tol, Mecit Suerdem

**Affiliations:** 1 Selcuk University, Selcuklu Faculty of Medicine, Department of Chest Diseases, Selçuklu, 42075, Konya, Turkey

**Keywords:** Bronchoscopy, Midazolam, Respiratory muscle strength

## Abstract

**Background:**

Flexible bronchoscopy (FB) is a procedure accepted to be safe in general, with low complication rates reported**.** On the other hand, it is known that patients with pre-existing respiratory failure have developed hypoventilation following FB. In this study the effects of FB on respiratory muscle strength were investigated by measuring maximum respiratory pressures.

**Methods:**

One hundred and forty patients, aged between 25 and 90 years, who had undergone diagnostic bronchoscopy between February 2012 and May 2012, were recruited to the study. Pre- and post-procedure maximal inspiratory pressure (MIP) and maximal expiratory pressure (MEP) were measured. A correlation between the MIP and MEP changes and patient characteristics and FB variables were investigated.

**Results:**

Significant decreases in both MIP and MEP values were observed following FB (p < 0.001 for both). Decreases were attributed to the midazolam used for sedation. Significant decreases in respiratory muscle strengths were observed especially in the high-dose midazolam group, compared to both low-dose and non-midazolam groups.

**Conclusions:**

It was determined that respiratory muscle weakness may arise post-procedure in patients who have undergone FB, and this is constitutively related to midazolam premedication. Respiratory muscle weakness might play a role in potential hypoventilation in critical patients who undergo FB.

## Background

Flexible bronchoscopy (FB) is a procedure that is widely used in the diagnosis and management of airway and lung diseases, and is accepted to be safe in general [1]. Data regarding bronchoscopy complications and low complication rates are mostly obtained from retrospective studies. However, the increase in therapeutic bronchoscopy applications, which are becoming more and more complex, and the bronchoscopy applications in risky patient groups cause an increase in complication rates [2].

Hypoventilation has been shown to develop during and after the procedure in patients who undergo bronchoscopy [3,4]. This has mostly been linked to sedative agents in relevant studies. However, to our knowledge, the effects of bronchoscopy itself and/or sedative agents on respiratory muscle strength have yet to be studied. The aim of this study was to evaluate the effects of bronchoscopy on respiratory muscle strength, which as an important role in respiratory function and induction of cough reflex [5,6].

## Methods

### Patients

One hundred and forty patients, aged between 25 and 90 years, who had undergone diagnostic bronchoscopy between February 2012 and May 2012 were recruited to the study. Patients with hemodynamic instability (heart rate below 50 bpm or above 120 bpm, systolic arterial blood pressure below 90 mmHg or above 180 mmHg), basal oxygen saturation <90%, PaCO_2_ > 45 mmHg, liver or kidney dysfunction, those who were diagnosed with a neuromuscular disease, had cancer with cachexia, medium (FEV_1_ between 50 and 80%), severe (FEV_1_ between 30 and 50%) or very severe (FEV_1_<30%) COPD, and those who could not cooperate with respiratory muscle strength test or with benzodiazepine allergy have not been included into the study. Study protocol was approved by Selcuk University Selcuklu Medical Faculty Ethics Committee. Written informed consent was obtained from all participants.

### Bronchoscopy

All bronchoscopy procedures were performed by the same experienced investigator in accordance with the international guidelines [7]. In the absence of any contraindications, IM atropine (0.01 mg/kg) was given to the patients half an hour prior to the procedure. 2% lidocaine was applied topically to the inside of the mouth, pharynx, upper respiratory airways and endobronchially. As a routine application at our clinic, IV midazolam was offered to all patients by the bronchoscopist prior to the procedure and 1 mg IV midazolam was given to those who accepted the offer 3 minutes prior to the procedure. While no additional doses were given to patients who had adequate sedation during the procedure, additional doses of 1 mg were given in 2-minute intervals to patients considered to be in need by the bronchoscopist. At the end of the study, patients were retrospectively separated into three groups: no midazolam (Group A), low-dose midazolam (Group B), and high-dose midazolam (Group C).

2–4 ml/min oxygen supplementation was performed in all patients, and oxygen flow was increased in case of hypoxemia. Flumazenil was kept available for suspected cases of respiratory depression. An IV vascular access was prepared, and electrocardiogram (ECG), oxygen saturation and non-invasive blood pressure monitoring were performed throughout the procedure. Total duration of bronchoscopy, midazolam dose and adverse events during the endoscopic procedure were recorded. Cardiopulmonary complications observed during the flexible bronchoscopy were defined as the following: hypotension (systolic blood pressure (BP) <90 mmHg or mean arterial blood pressure <60 mmHg), hypertension (systolic BP >180 mmHg or diastolic BP >90 mmHg), tachycardia (HR >100/min and/or a variation of >20% from baseline value), bradycardia (HR <50/min), and oxygen desaturation (SaO_2_ decrease <90% for >30s). Monitoring was continued for 2 hours following the procedure. 0–100 mm visual analogue scale (VAS) was used two hours after the flexible bronchoscopy to investigate the comfort of the procedure. A higher score meant better satisfaction or less discomfort (0: worst imaginable health state, 100: best imaginable health state).

### Maximum respiratory pressure measurements

A hand-held respiratory pressure meter (Micro RPM; Micro Medical, Chatham, UK) was used to measure respiratory muscle strength [8]. Maximal inspiratory pressure (MIP) was acquired from residual volume (RV) and the maximal expiratory pressure (MEP) was acquired from total lung capacity (TLC). Measurements were taken prior to bronchoscopy before premedication and after 30 minutes following the procedure by the same investigator without any knowledge of the clinical conditions of the patients. The operator explained the procedure before beginning and showed the correct manoeuvre. MIP was measured first, followed by MEP. Patients performed 5 maneuvers in intervals of 1 minute for each measurement and the highest values (<20% variance) were recorded [9].

### Statistical analyses

Descriptive statistics are shown as mean (±SD) or count (percentage). *T*-test was used to compare respiratory muscle strengths before and after bronchoscopy for all patients. *T*-test or One-Way ANOVA were used to compare each variable for the mean MIP and MEP changes. One-Way ANOVA and Chi-square tests were utilized in the comparison of continuous and categorical variables between various midazolam dose groups, respectively. The *post-hoc* comparisons of significant difference between the groups were performed using the Tukey test. The statistical significance level was set to 0.05.

## Results

Bronchoscopies were successfully performed in all of the 140 patients included in the study. As to the midazolam dosages: 34 patients did not want midazolam administration (Group A), 66 were administered 1 mg of midazolam prior to the procedure, and 40 received high doses (mean, 0.05 ± 0.03 mg/kg) of midazolam (Group C). Patient characteristics and complications developed during the bronchoscopy are presented in Table 1. The study population mostly comprised middle- and old-aged males. One third of the patients had mild COPD and the most common complication observed was tachycardia. On the contrary, hypertension, bradycardia, hypotension and desaturation were rarely encountered complications.

**Table 1:** Demographics and clinical and bronchoscopic characteristics of patients

**Variables**		**All patients (n = 140)**
Age, y		58.1 ±13.5
Male gender, n (%)		102 (72.9)
Patients with COPD, n (%)		44 (31.4)
History of smoking, n (%)		73 (52.1)
Smoking pack years		38.4 ±22.4
Duration of bronchoscopy (min)		15.9 ±6.1
Patient comfort (VAS mm)		78.8 ±17.1
Midazolam groups, n		
	Non	34
	Low-dose	66
	High-dose	40
Complications, n (%)		
	Tachycardia	62 (44.3)
	Bradycardia	2 (1.4)
	Hypotension	1 (0.7)
	Hypertension	13 (9.3)
	Desaturation	4 (2.9)

**Figure 1:**
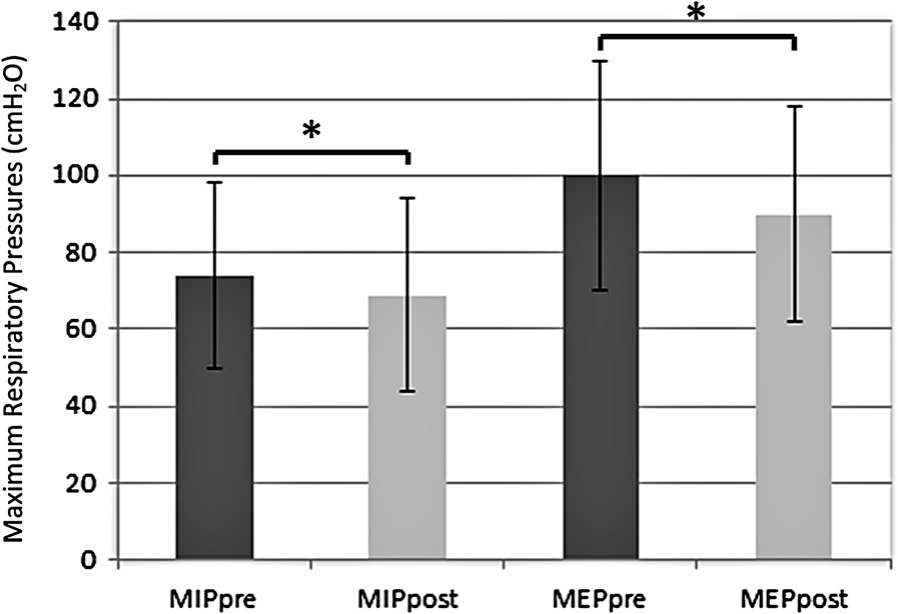
**Comparison of pre- and post-bronchoscopy respiratory muscle strength.** MIP, Maximum inspiratory pressure; MEP, Maximum expiratory pressure; *, p < 0.001.

When the overall pre- and post-procedure MIP and MEP values were compared (73.9 ±24.4 cmH_2_0 vs 68.9 ±25.2 cmH_2_0; 99.9 ±29.8 cmH_2_0 vs. 89.9 ±28.1 cmH_2_0 respectively), significant decreases in both values were observed (p < 0.001 for both comparisons; Figure 1).

Patients were dichotomized according to median age, median duration of bronchoscopy, smoking status, and COPD history in order to determine the variables that may cause a decrease in respiratory muscle strength. In addition, the pre- and post-procedure MIP and MEP values were investigated between the different midazolam dose groups as well. Among these variables, a significant difference was determined only in different midazolam dose groups in terms of Δ MIP and Δ MEP (Table 2).

**Table 2:** Comparison of the differences in pre- and post-procedure maximum respiratory pressures (Δ: pre-post) between different patient groups

	***N***	**Δ MIP (pre-post)**		**Δ MEP (pre-post)**	
		**Mean ± SD (cmH**_**2**_**O)**	**p**	**Mean ± SD (cmH**_**2**_**O)**	**p-value**
**Age (years)**					
<60	69	2.7 ±16.4	NS	10.0 ±22.61	NS
≥60	71	7.2 ±14.8		10.0 ±15.7	
**Smoking**					
Non-smoker	67	6.2 ±16.5	NS	9.8 ±14.8	NS
Smoker	73	3.9 ±14.9		10.2 ±22.7	
**COPD**					
No	96	5.4 ±15.7	NS	11.2 ±21.4	NS
Yes	44	4.2 ±15.9		7.4 ±13.5	
**Duration of bronchoscopy (min)**					
<15.5	69	4.5 ±16.6	NS	10.2 ±20.0	NS
≥15.5	71	5.5 ±15.0		9.9 ±18.8	
**Midazolam group**					
A	34	0.4 ±16.0	<0.001	6.2 ±16.0	<0.05
B	66	1.8 ±13.4		7.8 ±17.6	
C	40	14.2 ±15.6*		16.8 ±22.9**	

*Via *post-hoc* analysis, pair-wised comparison of Group C with both Group A and B (p < 0.001 for both). ** Via *post-hoc* analysis, pair-wised comparison of Group C with Group A (p = 0.04) and Group B (p = 0.05).

The post-hoc analysis in the midazolam groups revealed no significant difference between the Groups A and B in terms of Δ MIP (p > 0.05) while Δ MIP was higher in Group C when compared to Group A (p < 0.001) and Group B (p < 0.001). There was no significant difference between Group A and Group B in terms of Δ MEP (p > 0.05). Δ MEP was higher in Group C when compared to Groups A (p = 0.04) and B (p = 0.05).

The comparison of pre- and post-procedure MIP and MEP values among different midazolam dose groups using *T*-test revealed no significant difference in terms of MIP measurements in the Groups A and B; however, there was a significant decrease in post-procedure MEP values in both groups. Significant decreases in both the post-procedure MIP and MEP values were determined in Group C (Table 3).

**Table 3:** Comparison of pre- and post-flexible bronchoscopy MIP and MEP values in different midazolam dose groups

	**MIP (cm H**_**2**_**O)**		**p**	**MEP (cm H**_**2**_**O)**		**P**
	**Pre**	**Post**		**Pre**	**Post**	
**Group A**	80.5 ±25.2	80.0 ±24.7	NS	109.4 ±27.6	103.2 ±21	=0.03
**Group B**	69.3 ±23.1	67.6 ±22.8	NS	93.6 ±30.3	85.8 ±29.0	=0.001
**Group C**	75.9 ±24.9	61.7 ±26.6	<0.001	102.1 ±28.9	85.3 ±28.9	<0.001

No significant differences were determined in terms of age, sex, smoking status, ratio of patients with COPD, duration of bronchoscopy, and basal MIP/MEP measurements in different midazolam groups. However, there was a significant difference between the groups in terms of patient comfort assessed using VAS (p < 0.001), and while there was no significant difference between the Groups A and B in the *post-hoc* test, the VAS scores of Group C was determined to be higher than the patients in Group A and Group B (p < 0.001 for both comparisons; Table 4).

**Table 4:** Comparison regarding patient demographics, bronchoscopic characteristics and pre-procedure maximum respiratory pressures among different midazolam dose groups

	**Group A (n: 34)**	**Group B (n: 66)**	**Group C (n: 40)**	**p**
**Male gender, n (%)**	24 (70.6)	53 (80.3)	25 (62.5)	NS
**Age**	58.9 ±11.8	55.9 ±14.5	60.9 ±12.8	NS
**Patients with COPD, n (%)**	14 (41.2)	21 (31.8)	9 (22.5)	NS
**Smoking pack years**	27.6 ±11.6	38.9 ±18.9	36.9 ±21.9	NS
**Duration of bronchoscopy (min)**	15.5 ±5.9	15.6 ±5.4	16.9 ±7.3	NS
**Complications**				
Tachycardia	19	31	12	NS
Bradycardia	1	1	0	NS
Hypotension	0	1	0	NS
Hypertension	5	5	3	NS
Desaturation	2	1	1	NS
**Patient comfort**	76.2 ±17.4	74.4 ±17.4	88.1 ±12.5*	<0.001
**MIP 1 (cmH**_**2**_**O)**	80.5 ±25.2	69.3 ±23.1	75.9 ±24.9	NS
**MEP 1**	109.4 ±27.6	93.6 ±30.1	102.1 ±28.9	NS

* When compared to Groups A and B using the post-hoc test: p < 0.001.

## Discussion

In this study, where -to our knowledge- the effects of FB and premedication on respiratory muscle strengths were investigated for the first time, it was revealed that midazolam, administered for sedation purposes in bronchoscopy, might affect negatively the respiratory muscle strengths, as shown with MIP and MEP measurements.

Flexible bronchoscopy is generally regarded as a safe procedure [1]. Hypoventilation is one of the most common complications and many causes of hypoventilation related to FB have been defined. These include ventilation-perfusion mismatch related to the procedure itself, upper airway obstruction, sedation-related central respiratory depression, and increased resistance due to introduction of bronchoscope into the trachea [3,10]. Our findings show that sedation-related respiratory muscle weakness can be included among these mechanisms.

Pulse oxymetry is utilized in many centers for monitoring hypoventilation during bronchoscopy. On the other hand, patients are routinely given oxygen during FB in many centers. Bronchoscopy guidelines recommend oxygen supplementation to maintain oxygen saturation at a minimum of 90%, because it will reduce the risk of arrhythmia during and after bronchoscopy [7]. However, even though severe CO_2_ retention may occur during the procedure, oxyhemoglobin desaturation may not be observed. Chhajed et al*.*[3] have performed cutaneous carbon dioxide tension (PcCO_2_) measurements in addition to the oxymetry in patients that underwent FB in which sedation was achieved with intermittent intravenous midazolam and 5 mg of hydrocodone, and determined an increase in PcCO_2_ in all but one patient. The highest PcCO_2_ value was significantly associated to the baseline PcCO_2_ (p < 0.0001) and lowest SpO_2_ (p = 0.016). Dreher et al*.*[4] have utilized PcCO_2_ measurements in order to evaluate alveolar hypoventilation in patients with pre-existing respiratory failure, and determine a significant increase in PcCO_2_ during the procedure. In their study, while no significant difference was determined in terms of PcCO_2_ during FB between the groups where the patients were sedated with either midazolam alone or midazolam plus alfentanil, PcCO_2_ was higher compared to baseline in the midazolam alone group 120 minutes after the procedure than the midazolam plus alfentanil group. The cause for prolonged hypoventilation was considered to be midazolam due to the fact that the midazolam alone group received twice the amount of midazolam than the midazolam plus alfentanil group (4 mg vs. 2 mg). We believe that the decrease in both the inspiratory and expiratory respiratory muscle strengths, which was determined in the high-dose (0.05 ± 0.03 mg/kg) midazolam group in our study, might play an important role in hypoventilation that has been determined in the two studies mentioned above. However, because we did not measure PcCO_2_ in our study, we do not know whether there was an increase in the CO_2_ values or not, and thus, we do not know whether the determined muscle weakness has any clinical importance or not.

The effects of midazolam on respiratory muscle strength have been previously shown in experimental and clinical studies. Fujii et al. [11] have investigated the effects of midazolam and propofol on diaphragm contractility in dogs. In that study, they have induced diaphragmatic fatigue with intermittent supramaximal bilateral electrophrenic stimulation at low or high frequency (20 and 100 Hz, respectively), and after the induction of fatigue, in order to assess the diaphragm contractility, transdiaphragmatic pressure (Pdi) and integrated electrical activity of the crural (E_di-cru_) and costal (E_di-cost_) parts of the diaphragm were measured. Their findings showed that midazolam caused a decrease in Pdi at both frequencies when compared to fatigued values (p < 0.05), and that E_di-cru_ and E_di-cost_ at 100 Hz stimulation during midazolam administration were below the baseline values (p < 0.05). They have also reported a lower Pdi value in the midazolam group than in the propofol group (p < 0.05).

In another study, the effects of sedative (0.1 mg/kg/h) and anesthetic (0.5 mg/kg/h) dosages of midazolam on the decrease in diaphragm contractility, fatigue (detail fatigue rating [DFR]), have been evaluated in dogs [12]. They showed that an infusion of midazolam has caused a decrease from baseline values (p < 0.05) in Pdi at 20 and 100 Hz stimulations, and that%E_di-cru_ and % E_di-cost_ values at 100 Hz were below baseline (p < 0.05) in both sedative and anesthetic groups. They have also demonstrated that the Pdi and % E_di_ decrease was greater in the anesthetic dose group than in the sedative dose group (p < 0.05). Their findings show that contractility of fatigued diaphragm dose-dependently decreases with midazolam.

Molliex et al*.*[13] have studied the effects of midazolam, with a dosage of 0.1 mg/kg, on total pulmonary resistance and diaphragmatic, intercostal and abdominal muscle patterns in 9 healthy volunteers. Changes in gastric pressure (ΔPga) and pleural pressure (ΔPpl) were measured in all participants, and the reduction in diaphragm contractility was evaluated with ΔPga/ ΔPpl. Midazolam was determined to increase total pulmonary resistance in sedative doses, and associatively, an increase in intercostal muscle activity was also determined; however, the diaphragmatic contribution to respiratory process was found to be decreased. This was explained as a shift from an abdominal breathing to predominantly rib cage breathing rather than a decrease in diaphragm contractility. Even though upper airway measurements were not taken in the current study, the increase in pulmonary resistance is thought to be associated with upper airway occlusion. Diazepam causing a decrease in the activity of the genioglossus muscle, which has an important role in maintaining the patency of the upper airway, supports this hypothesis [14].

It is known that bronchoscopy deleteriously affects pulmonary mechanics and lung volumes [10,15]. This may arise a question regarding whether the significant reductions in maximal pressures are related to lung volumes rather than muscle weakness or respiratory muscle strength. MIP is measured at or close to RV and MEP at or close to TLC. Sometimes these measurements were performed at functional residual capacity. Although the latter may be more accurate for some studies, in that case the lung volumes should be specifically stated [16]. In patients with abnormally high lung volumes, a low MIP may partly reflect the shortened inspiratory muscle fiber length associated with increased lung volume at RV rather than reduced inspiratory muscle strength; however, in our study all moderate to very severe COPD patients were not taken into the study and also the groups were not different regarding the number of mild COPD patients. Therefore it may be considered that the reductions in maximal pressures are probably not related to lung volumes.

Although there is limited falls in MEP values in patients who were not sedated during bronchoscopy, when Δ MEP values were compared between groups, the *post-hoc* analysis revealed that the difference was statistically significant only with group A and C and the decline in MEP values was higher in the high-dose midazolam group. When pre and post MEP values within each group were compared, post MEP values in group B and C were significantly lower than group A. It is notable that although post MIP values were significantly lower only in Group C, there were remarkable falls in post MEP values in all groups. The fall in post MEP values even in group A, i.e. in groups who were not sedated with midazolam might be due to exhaustion of the patient after repeated maneuvers for correct MIP measurements.

Even though the overall bronchoscopy comfort scores were quite high in our study, the fact of significantly higher VAS scores of the patients in the high-dose rather than in the low-dose midazolam group, and of the similarity of the patient comfort levels in the low-dose and no midazolam groups can be explained by the bronchoscopist’s tendency to administer low doses. It is known that objective techniques, such as electroencephalogram-based bispectral index [17] or Ramsay sedation score [18], allow a more effective titration of the sedatives. In addition, it is also shown that both inter-individual and intra-individual variations are seen in the online sedation monitoring in healthy volunteers who were sedated with midazolam [19]. This condition may explain why some patients needed high doses and some low doses of midazolam in our study although the bronchoscopist decided the appropriate sedation level in patients subjectively. In humans, midazolam is mostly metabolized by CYP3A4⁄5 isozymes to one major metabolite, 1-hydroxymidazolam and to some extent to 4-hydroxymidazolam and 1,4-dihydroxymidazolam [20,21]. It is well known that wide inter-individual variations in hepatic and intestinal CYP3A activity are seen in the human population [22].

## Conclusion

In conclusion, midazolam for premedication purposes has been determined to increase comfort in patients undergoing flexible bronchoscopy, however, causing significant decrease in respiratory muscle strength. This might increase the probability of possible complications following bronchoscopy in critical patients who have advanced COPD with limited respiratory reserves or respiratory insufficiency. Certainly there is a special need for further studies with risk patient groups and the use of high-dose midazolam.

## Competing interests

The authors of the paper ”Flexible bronchoscopy may decrease respiratory muscle strength: premedicational midazolam in focus” declare to have no competing interests that might be related to the contents of the manuscript.

## Authors’ contribution

BT helped design the study, conduct of the study, collection of data, analysis of data and prepare the manuscript. FK helped conduct of the study, collection of data, analysis of data and prepare the manuscript. ST helped conduct of the study, collection of data. MS helped conduct of the study, collection of data, and prepare the manuscript. All authors read and approved the final manuscript.
